# Expression of TDRD9 in a subset of lung carcinomas by CpG island hypomethylation protects from DNA damage

**DOI:** 10.18632/oncotarget.22709

**Published:** 2017-11-27

**Authors:** Macarena Guijo, María Ceballos-Chávez, Elena Gómez-Marín, Laura Basurto-Cayuela, José C. Reyes

**Affiliations:** ^1^ Centro Andaluz de Biología Molecular y Medicina Regenerativa-CABIMER, Consejo Superior de Investigaciones Científicas- Universidad de Sevilla-Universidad Pablo de Olavide, Seville, Spain

**Keywords:** DNA methylation, piRNA, DNA damage, DNA-PK, cancer

## Abstract

Tudor domain containing protein 9 (TDRD9) is a RNA helicase normally expressed in the germline, where it is involved in the biosynthesis of PIWI-interacting RNAs (piRNAs). Here, we show that *TDRD9* is highly expressed in a subset of non-small cell lung carcinomas and derived cell lines by hypomethylation of its CpG island. Furthermore, *TDRD9* expression is associated with poor prognosis in lung adenocarcinoma. We find that downregulation of *TDRD9* expression in TDRD9-positive cell lines causes a decrease in cell proliferation, S-phase cell cycle arrest, and apoptosis. Transcriptomic analysis demonstrated that *TDRD9* knockdown causes upregulation of cell cycle and DNA repair genes. We also observed that *TDRD9* knockdown triggers activation of the catalytic subunit of the DNA dependent protein kinase (DNA-PKcs) and phosphorylation of H2A.X, which are indicative of an increase of DNA double strand breaks. *TDRD9*-silenced cells also presented aberrant mitosis and abnormal-shaped nuclei indicating defects in chromosomal segregation. Finally, *TDRD9* silencing caused hypersensitivity to the replication stress inducer aphidicolin, while overexpression of the protein increased resistance to the drug, suggesting that TDRD9 protects from replicative stress to TDRD9-positive tumor cells. Thus, our results place TDRD9 as a marker for prognosis and as a potential therapeutic target in a subset of lung carcinomas.

## INTRODUCTION

Tudor domain containing protein 9 (TDRD9) is a putative ATP-dependent DEXH-box RNA helicase that contains a TUDOR domain [1, 2]. Under non-pathological conditions, human *TDRD9* is predominantly expressed in the germline. Mice Tdrd9 has been detected in spermatogonia, spermatocytes, spermatids in the testis and oocytes in the ovary [2]. Mammalian TDRD9 and the *Drosophila* homologue Spindle-E are involved in the process of biogenesis of a conserved class of small RNAs, called piRNA for their association with PIWI proteins [2–7]. piRNAs are implicated in transposon silencing at the transcriptional level in germ cells [5, 8–10], as recently reviewed in [11]. In fact, Tdrd9 is essential for silencing *Line-1* retrotransposons in the mouse male germline [2]. *Tdrd9* knockout mice display male sterility, probably as a consequence of massive *Line-1* activation. *Line-1* DNA is hypomethylated in *Tdrd9*^-/-^ mutants, indicating a connection between piRNA-mediated transcriptional silencing and DNA methylation [2, 9]. However, the specific role of TDRD9 in piRNA biogenesis and *Line-1* DNA methylation is unknown. TDRD9 interacts with MIWI2 (one of the mouse PIWI proteins) through its TUDOR domain and has been localized both in the nuclei and in the cytoplasm in germ cells-specific RNA structures called *nuage* [1, 2, 12].

Expression of germline-specific factors in tumor cells has received increased attention during the last years due to the fact that they can be ideal targets for cancer therapy [13–15]. Thus, the germline-restricted expression of these factors should, presumably, reduce side effects. As nothing was known about the role of TDRD9 in cancer, therefore we decided to search for abnormal *TDRD9* expression levels in different cancer databases and cell lines. Here we show that the *TDRD9* gene is expressed in about 15% of lung adenocarcinomas and 30% of skin melanoma tumors, but not in the normal tissues. The *TDRD9* gene presents a CpG island in its 5´ region that is hypomethylated in spermatic tissues but hypermethylated in most human tissues where the gene is not expressed. We show that TDRD9-expressing cell lines and tumor samples display a strong hypomethylation of the *TDRD9* CpG island. Silencing of *TDRD9* in lung cancer cell lines that express the gene causes a S-phase cell cycle arrest and increased DNA damage. Furthermore, TDRD9-deficient cells exhibit hypersensitivity to the replication stress inducer aphidicolin, while TDRD9 overexpression causes resistance to the drug, suggesting that TDRD9 plays a role in protection from replicative stress.

## RESULTS

### TDRD9 is highly expressed in lung adenocarcinoma and skin melanoma

**Figure 1:**
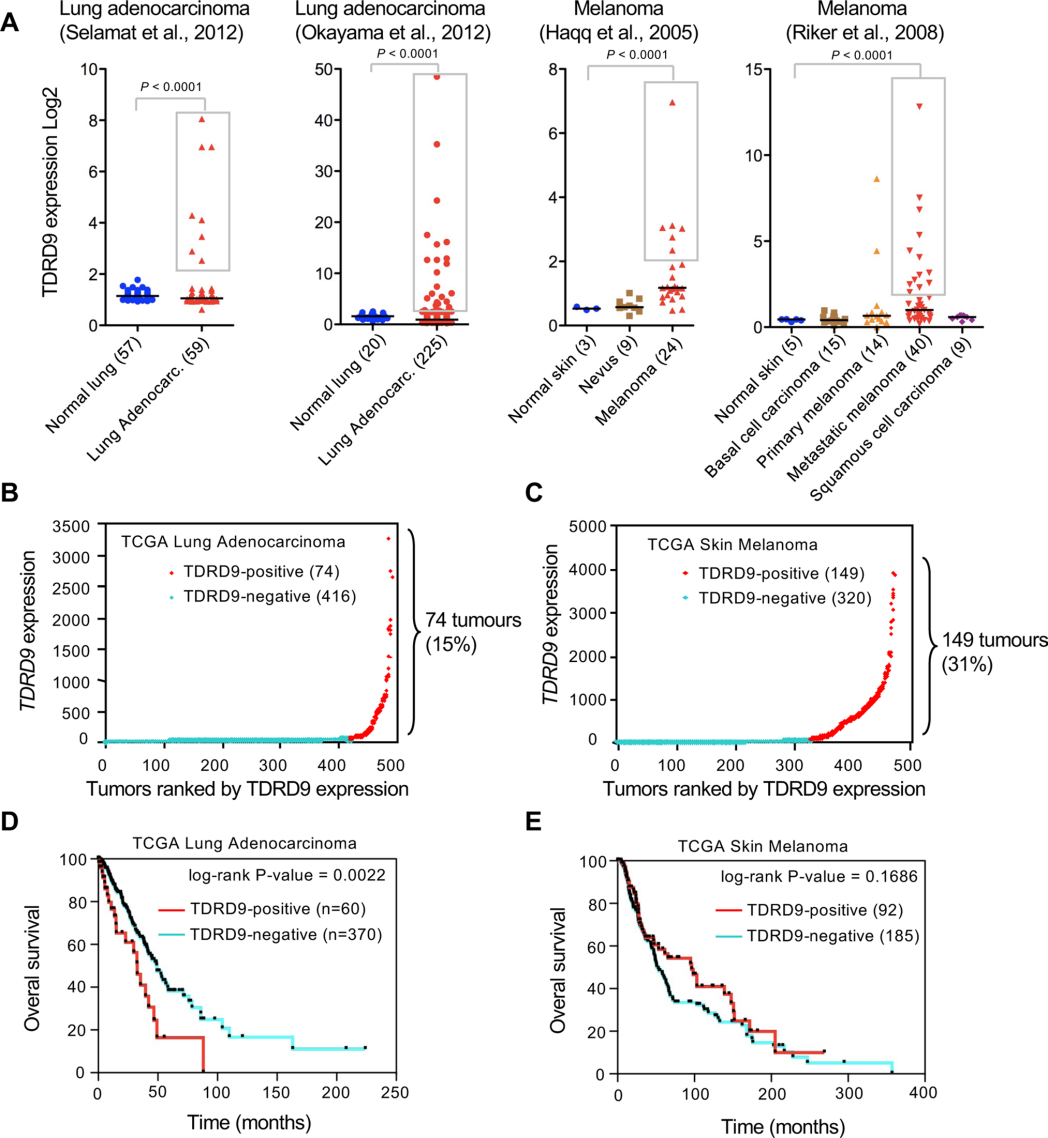
*TDRD9* expression in lung adenocarcinoma and skin melanoma (**A**) Analysis of *TDRD9* expression in lung adenocarcinoma and skin melanoma. Data were obtained from the indicated sources through ONCOMINE. Statistical significance value for Student’s *t*-test is *P* < 0.0001. (**B**, **C**) RNA-seq expression data (RSEM normalized) from lung adenocarcinoma (TCGA-LUAD) (B) and skin melanoma (TCGA-SKCM) cohorts (**C**). (**D**, **E**) Kaplan-Meier survival plots for TCGA-LUAD patients (D) and TCGA-SKCM patients (E). *P* values were calculated by log-rank test. Data obtained from The Cancer Genome Atlas (TCGA). (**B**-**E**) TDRD9-positive tumors are shown in red; TDRD9-negative tumors are shown in blue.

As a first step to investigate a possible role of TDRD9 in cancer we explored publicly available cancer gene expression sets using the ONCOMINE database [16]. Analysis of *TDRD9* expression in two sets of lung adenocarcinomas [17, 18] and skin melanomas [19, 20] showed that, in the cancer samples, values did not follow a normal (Gaussian) distribution (Shapiro-Wilk test *P* < 0.00001) (Figure 1A), suggesting that the positive outlier values did not result from random variance. In fact, *TDRD9* expression was significantly increased (more than 2-fold the median) in 13% (8 out of 59 samples) to 15% (34 out of 225) of the analyzed lung adenocarcinoma samples (*P* < 0.0001 with respect to the level in normal lung tissue). Similarly, *TDRD9* expression is increased in 25% (6 out of 24) to 32% (13 out of 40) of the melanoma samples (*P* < 0.0001 with respect to the level in normal skin) (Figure 1A). We have also analyzed RNA-seq expression data from lung adenocarcinoma (TCGA-LUAD) [21] and skin melanoma (TCGA-SKCM) [22] cohorts from The Cancer Genome Atlas (TCGA) through cBioPortal [23]. The majority of TCGA-LUAD tumors did not express, or expressed at very low levels, the *TDRD9* transcript (TDRD9-negative tumors) (Figure 1B). However, 74 out of 491 tumors (15%) displayed high levels (≥ 5-fold over the median) of *TDRD9* transcript (TDRD9-positive tumors). Similarly, most TCGA-SKCM samples either did not express *TDRD9* or expressed it at very low levels. However, 149 of the 471 tumors (31%) expressed *TDRD9* at high levels (≥ 5-fold over the median) (Figure 1C). Therefore, our analysis indicates that *TDRD9* is highly expressed in a subset of lung adenocarcinoma and skin melanoma tumors. Similar analysis of other types of tumors from TCGA showed that *TDRD9* is expressed at high levels in a variable proportion of tumors, ranging from 1% in the case of colon cancer to 50% in acute myeloid tumors (AML) (Supplementary Figure 1).

Kaplan-Meier survival curves revealed that TCGA-LUAD patients with TDRD9-positive tumors (*n* = 60) had a shorter survival as compared to those with TDRD9-negative tumors (*n* = 370), with median survivals of 32.7 versus 49.2 months, respectively (log-rank *P* = 0.0022) (Figure 1D). In contrast, no significant survival differences were observed between TCGA-SKCM patients with positive or negative TDRD9 tumors (*P* = 0.1686) (Figure 1E). These data indicate that *TDRD9* expression is a prognostic marker for a subset of lung adenocarcinoma.

**Figure 2:**
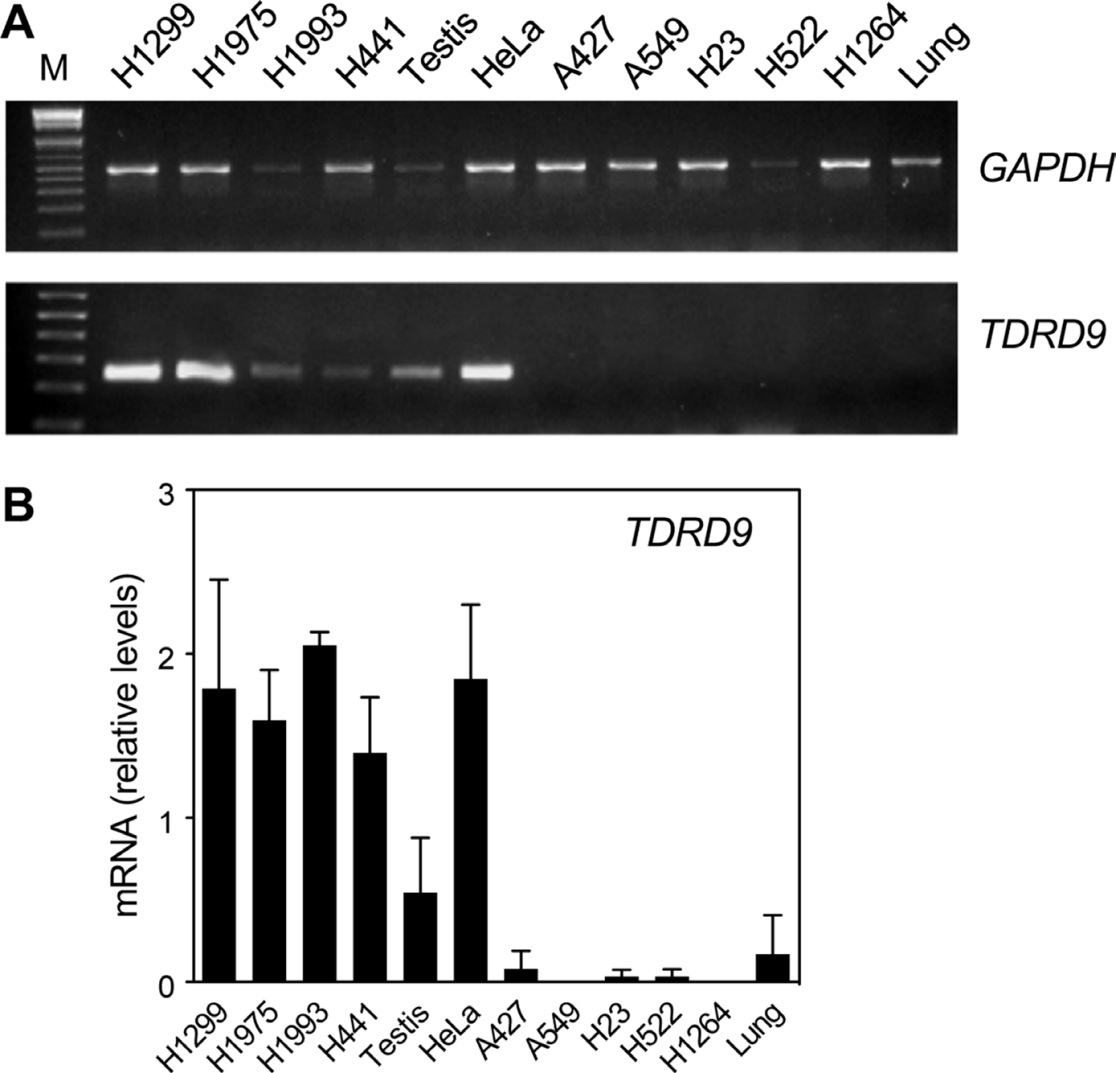
*TDRD9* is expressed in a subset of lung carcinoma cell lines Several non-small-cell lung carcinoma (NSCLC) (NCI-H1299, NCI-H1975, NCI-H23, NCI-H522) and lung adenocarcinoma (LA) cell lines (NCI-H1993, NCI-H441, A549, A427, NCI-H1264) as well as HeLa cells were used to determine the expression of *TDRD9* by conventional RT-PCR (**A**) or by RT-qPCR (**B**). Normal testis and lung tissues were used as positive and negative controls, respectively. M: 1 Kb Plus ladder. Values were normalized to *GAPDH* amplification. Data are the mean of at least *n* = 6 qPCR reactions from three independent experiments. Error bars represent ± SD values.

Next we analyzed *TDRD9* expression by conventional (RT-PCR) and quantitative reverse transcription-PCR (RT-qPCR) in lung adenocarcinoma (LA) cell lines and other non-small-cell lung carcinoma (NSCLC) cell lines. Four of the cell lines analyzed—the NSCLC lines NCI-H1299 and NCI-H1975, and the LA lines NCI-H1993 and NCI-H441—showed levels of *TDRD9* mRNA similar to those in testis tissue (Figure 2A and 2B). Previous data from our lab indicated that *TDRD9* is also expressed in the cervix carcinoma cell line HeLa, therefore we also include this cell line in our assessment, as a positive control. In contrast, five cell lines—the LA lines A549, A427, and NCI-H1264, and the NSCLC lines NCI-H23 and NCI-H522—did not show significant levels of *TDRD9* mRNA. Human lung tissue also had very low levels of *TDRD9* expression (Figure 2A and 2B). These data confirm that *TDRD9* is expressed in a subset of lung carcinoma cell lines.

### The *TDRD9* CpG island is hypomethylated in a subset of lung cancer

We next investigated the origin of the *TDRD9* expression in tumors. First, we verified that *TDRD9* expression was not correlated to the number of copies of the *TDRD9* gene in the TCGA-LUAD and TCGA-SKCM tumors, ruling out gene amplification as the reason for high *TDRD9* expression (Supplementary Figure 2).

**Figure 3:**
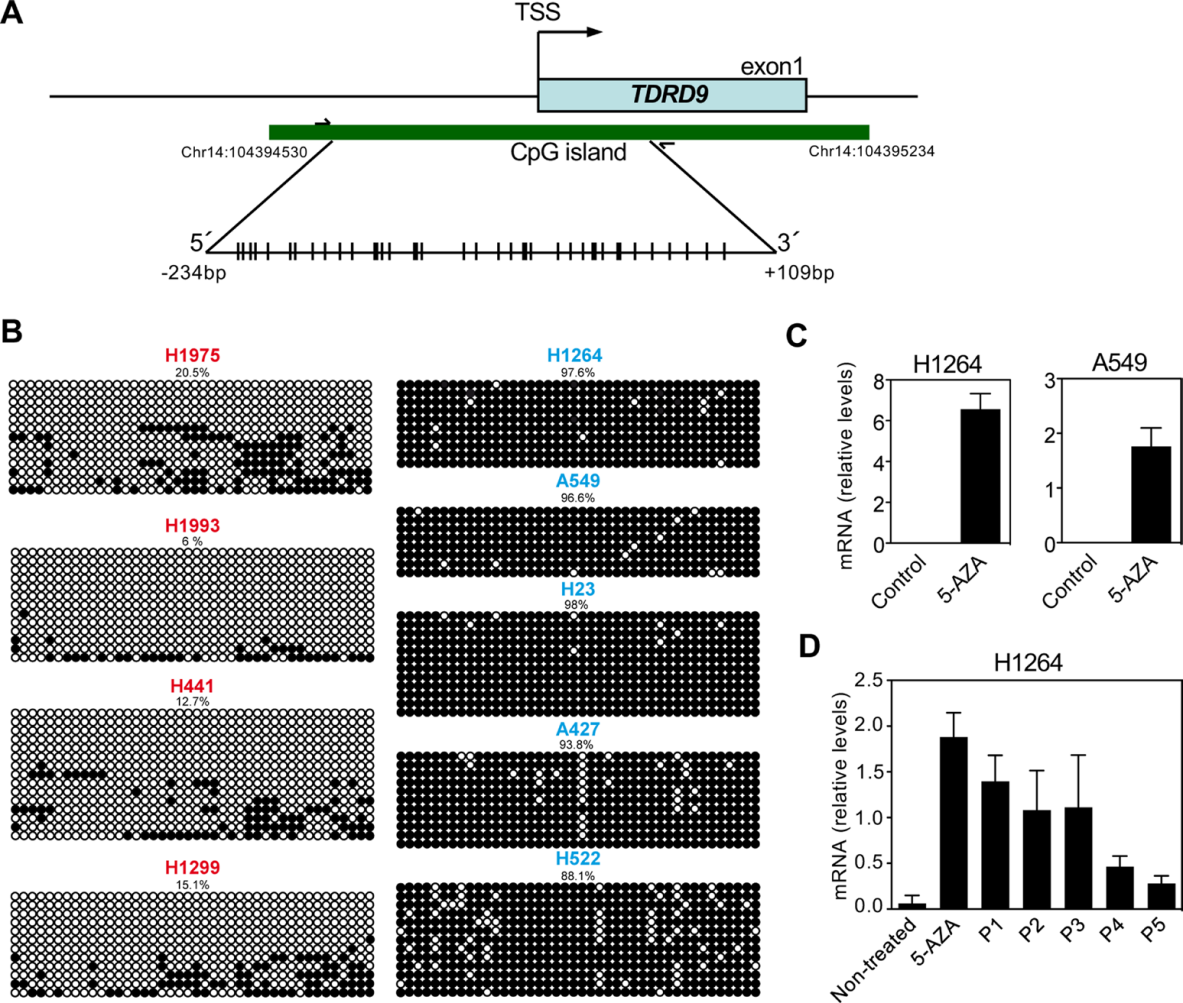
DNA methylation profile of the CpG island of *TDRD9* promoter (**A**) Schematic diagram of the CpG island of *TDRD9* promoter (-287 to +417 with respect to the transcription start site (TSS)). Amplified region for determination of DNA methylation (-234 to +109) is also shown. CpG pairs are represented by sticks. (**B**) Determination of DNA methylation by bisulfite conversion and sequencing of a region (-234 to +109 respect to the TSS) of the *TDRD9* CpG island from different lung carcinoma cell lines. Open circles denote unmethylated CpGs, and filled circles represent methylated CpGs. Eight to fourteen independent clones of each sample were sequenced. (**C**) Relative mRNA expression levels of *TDRD9* gene by RT-qPCR in TDRD9-negative lung carcinoma cell lines (H1264 and A549) after the treatment with 5-aza-2´-deoxycytidine for 72 hours. (**D**) Re-silencing of *TDRD9* gene expression in the H1264 cell line following of 5-aza-2´-deoxycytidine removal. Cells were treated with 5-aza-2´-deoxycytidine for 72 hours and then washed and cultured in the absence of the drug. Cells were passed 1:3 dilution every three days, and samples were taken at the indicated passage (P) for RNA isolation. Data are the mean of at least *n* = 6 qPCR reactions from three independent experiments. Error bars represent ± SD values.

The *TDRD9* gene has a promoter-associated CpG island that expands from nucleotides –287 to +417 with respect to the transcription start site (TSS) (Figure 3A). Analysis of data from the Roadmap Consortium [24] by using the UCSC Genome Browser indicated that the *TDRD9* CpG island is hypermethylated in most human organs (Supplementary Figure 3), including lung, consistent with the absence of expression of *TDRD9* in these organs. However, the region was hypomethylated in spermatozoa, where *TDRD9* is expressed. Then, we investigated the DNA methylation status of a region of the CpG island around the TSS (–234 to +109), in lung cancer cell lines with different levels of expression of *TDRD9* using bisulphite genomic sequencing analyses of multiple clones (Figure 3B). The *TDRD9* CpG island was hypermethylated in the cell lines that did not express *TDRD9* (A549, A427, H522, H1264 and H23). However, a strong hypomethylation of the CpG island was observed in the cell lines that expressed *TDRD9* (H1299, H1975, H1993 and H441). These data indicate that *TDRD9* expression in LA and other types of NSCLC is associated to promoter CpG island hypomethylation. Consistently, treatment of H1264 and A549 cells that do not express *TDRD9,* and that display *TDRD9* CpG island hypermethylation, with the DNA-demethylating agent 5-aza-2´-deoxycytidine promoted *TDRD9* expression (Figure 3C). Interestingly, *TDRD9* expression was progressively lost in the H1264 cell population upon 5-aza-2´-deoxycytidine removal, suggesting that signals that determine *TDRD9* CpG island methylation are still present or, alternatively, that *TDRD9* expression is deleterious for the proliferation in this cell line (Figure 3D).

Analyzing the *TDRD9* methylation status in the TCGA-LUAD tumors revealed that *TDRD9* hypomethylation was associated with transcript overexpression (Spearman correlation test, *ρ* = –0.44, *P* < 0.00001, *n* = 429) (Supplementary Figure 4A). In fact, most (98%) of the tumor samples that did not express *TDRD9* showed a high level of *TDRD9* methylation (fraction of methylated cytosines > 0.5). In contrast, 83% of the samples that expressed *TDRD9* displayed a low level of *TDRD9* methylation (fraction of methylated cytosines < 0.5). Similar results were obtained from TCGA-SKCM (*ρ* = –0.54, *P* < 0.00001, *n* = 458), where 87% of the samples that expressed *TDRD9* showed a low level of *TDRD9* methylation (fraction of methylated cytosines < 0.5) (Supplementary Figure 4B). It has been shown that cancer CpG island hypomethylation often occurs in large genomic domains [25, 26]. Therefore, we analyzed the methylation status of the genes neighboring *TDRD9* (Supplementary Figure 4C) by dividing the samples into two categories: TDRD9-positive and TDRD9-negative tumors, as defined in Figure 1B and 1C. While the level of DNA methylation of *TDRD9* clearly decreased in TDRD9-positive tumors with respect to TDRD9-negative tumors (*P* < 0.0001), the methylation status of the genes neighboring *TDRD9* was independent of the level of *TDRD9* expression (Supplementary Figure 4D and 4E). These data indicate that hypomethylation of *TDRD9* is not linked to a general alteration of DNA methylation at the *TDRD9* genomic region.

### TDRD9 knockdown impairs proliferation in TDRD9-positive cell lines

**Figure 4:**
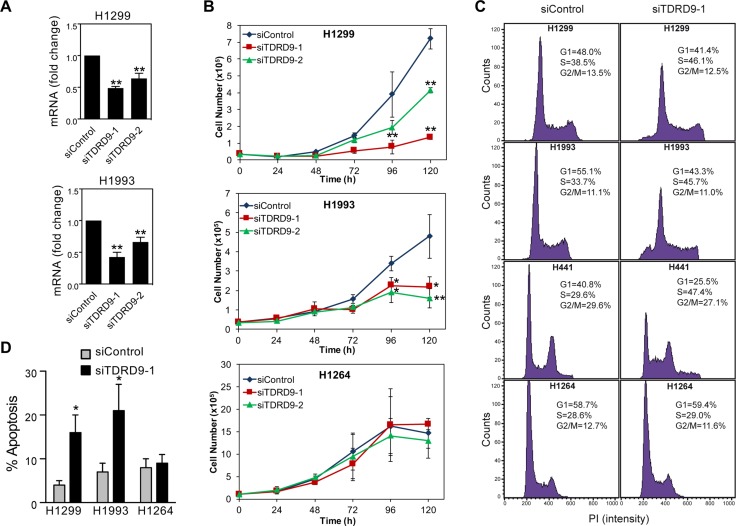
*TDRD9* depletion impairs proliferation in TDRD9-positive cell lines (**A**) Expression levels of *TDRD9* in H1299 and H1993 cell lines after the transfection with siRNA control or two different siRNAs against *TDRD9*. Data are the mean of at least *n* = 6 qPCR reactions from three independent experiments. Error bars represent ± SD values. (**B**) Growth curve of TDRD9-depleted cell lines. A TDRD9-negative lung carcinoma cell line (H1264) was used as a control. Data are the average of four (H1299 and H1993) or three (H1264) independent experiments. Error bars represent standard deviation. Levels of *TDRD9* mRNA during time points of the growth curve is shown in Supplementary Figure 5. (**C**) Flow cytometry of TDRD9-positive (H1299, H1993, and H441) or TDRD9-negative (H1264) lung carcinoma cell lines 72 hours after transfection of control siRNA or siRNA against *TDRD9*. Representative experiments are shown. (**D**) Level of apoptosis was determined 72 hours after transfection of control siRNA or siRNA against *TDRD9,* by measuring the percentage of cells containing a subG1 DNA content by flow cytometry. Data are the average of three independent experiments. Error bars represent ± SD values. **A**, **B**, **D**. Significance respect to the siControl was tested by using Student’s *t*-test. ^*^P < 0.05; ^**^P < 0.01.

Having observed the CpG island hypomethylation-associated *TDRD9* expression in a number of lung cancer cell lines (TDRD9-positive), we decided to investigate the role of TDRD9 in proliferation of these cells. Transfection of two different siRNAs that target the *TDRD9* transcript (siTDRD9-1 and siTDRD9-2) significantly impaired cell proliferation in the H1299 and H1993 cell lines as compared to cells transfected with a control siRNA (Figure 4A and 4B; Supplementary Figure 5). As a control, we also verified that *TDRD9* knockdown did not affect proliferation of the TDRD9-negative H1264 cell line (Figure 4B). We then analyzed the cell cycle of TDRD9-depleted cells by flow-cytometry. *TDRD9* knockdown provokes a reduction of the percentage of cells in G1 phase, and a concomitant increase of the percentage of cells in S phase, in the TDRD9-positive cell lines H1299, H1993, and H441, but not in the TDRD9-negative H1264 (Figure 4C), suggesting that the lower proliferation observed upon *TDRD9* knockdown can be due to S phase arrest. In addition, siTDRD9-1 transfection caused a 3- to 4-fold increase in the percentage of apoptotic cells after 72 hours in the TDRD9-positive cell lines H1299 and H1993, but not in the TDRD9-negative cell line H1264 (Figure 4D). Next we investigated the effect of lentiviral-mediated TDRD9 overexpression in cell proliferation of H1299, H1993 and H1264 cells. Overexpression of *TDRD9* caused a small but repetitive increase of proliferation in the TDRD9-positive cell line H1299 but had no effect in H1993 or in the TDRD9-negative H1264 (Supplementary Figure 6).

### Characterization of transcriptional changes caused by *TDRD9* knockdown

It has been shown that a complex between Tdrd9 and Miwi2 is involved in controlling *Line-1* retrotransposase expression through piRNA-mediated hypermethylation in mouse germ cells [2]. In fact, *PIWIL4* gene (the human orthologue of *Miwi2*) was expressed in several of the cell lines analyzed (Supplementary Figure 7A). Then, we determined the level of expression of the *LINE-1* transcript in cell lines expressing or not expressing *TDRD9* and *PIWIL4*. No correlation was present between *TDRD9* and/or *PIWIL4* expression and the level of expression of the *LINE-1* transcript (Supplementary Figure 7B and C). We next showed that downregulation of *TDRD9* expression in TDRD9-positive and PIWIL4-positive cell lines did not affect the level of *LINE-1* transcript (Supplementary Figure 7D). In conclusion, these data suggest that TDRD9 does not control *LINE-1* transcript expression in lung cancer cells.

**Figure 5:**
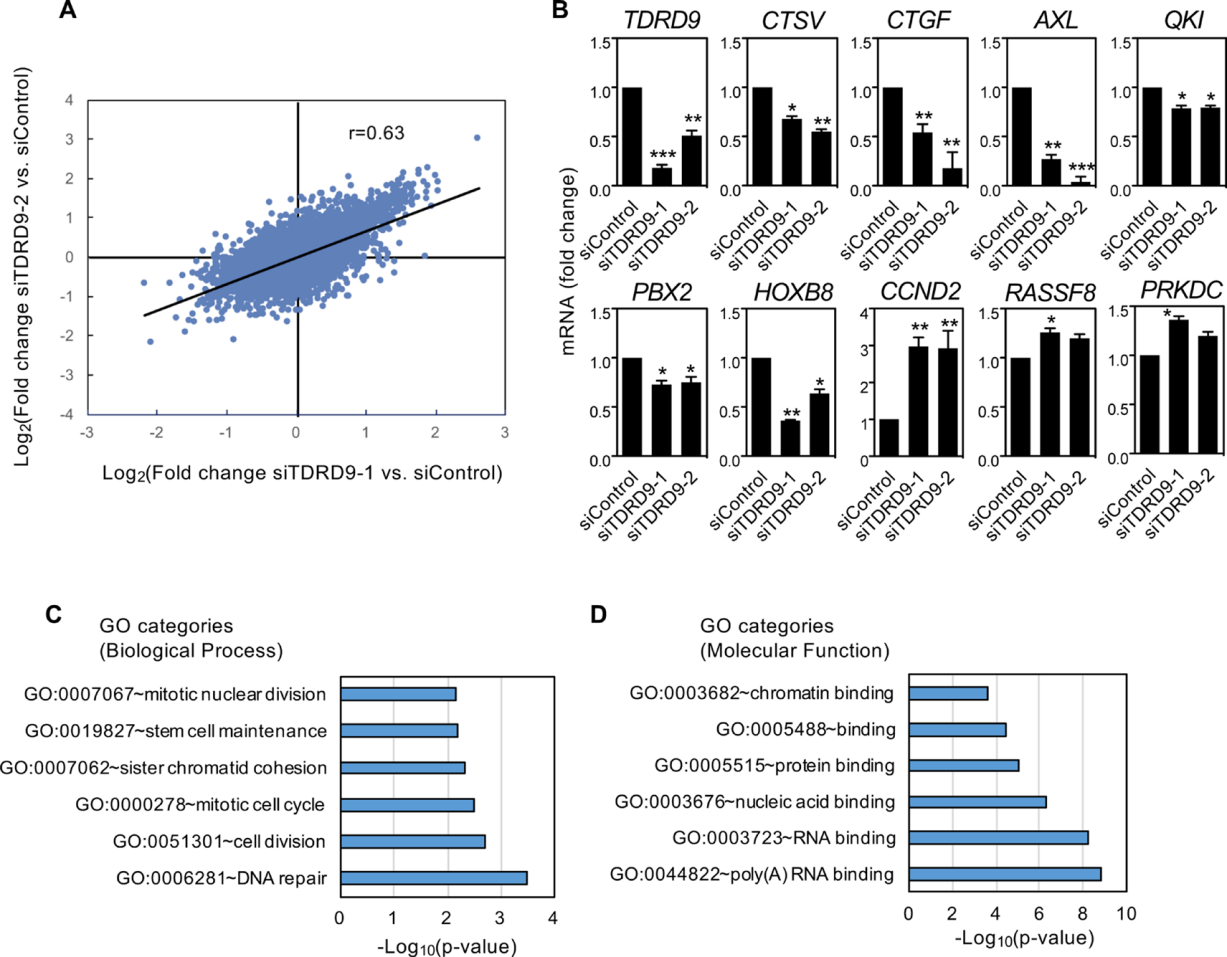
Characterization of transcriptional changes caused by *TDRD9* knockdown in the lung adenocarcinoma cell line H1993 (**A**) Correlation between the results obtained after knockdown of *TDRD9* using two different siRNAs (Pearson coefficient, *r* = 0.634; *P* < 0.0001). (**B**) Effect of *TDRD9* knockdown on the expression of the indicated genes. Expression levels of *TDRD9* were determined as a control. mRNA levels were determined by RT-qPCR. Data are the mean of at least *n* = 6 qPCR reactions from three independent experiments. Error bars represent ± SD values. Significance respect to the siControl was tested by using Student’s *t*-test. ^*^P < 0.05; ^**^P < 0.01; ^***^P < 0.0001. (**C**) Biological process-GO categories enriched in genes upregulated after *TDRD9* knockdown. (**D**) Molecular function–GO categories enriched in genes upregulated after *TDRD9* knockdown. Bonferroni-corrected *P* values were -log10 transformed.

To elucidate the transcriptional consequences of TDRD9 depletion in a TDRD9-positive cell line, we performed RNA expression profiling using Affymetrix DNA microarrays of H1993 cells transfected with siTDRD9-1, siTDRD9-2, or control siRNA. The two independent siRNAs against *TDRD9* produced similar transcriptional changes with respect to the control siRNA (Pearson coefficient, *r* = 0.634, *P* < 0.0001) (Figure 5A). We selected 138 genes that were differentially expressed by both siRNAs (FDR < 0.01 and |lineal fold change| ≥ 2 fold) of which 123 were up-regulated and 15 were down-regulated (Supplementary Table 1). Misregulated expression of nine genes upon reduction of *TDRD9* expression was confirmed by RT-qPCR (Figure 5B).

Biological process-Gene Ontology (BP-GO) analysis showed that several functional categories related to DNA repair, cell cycle, and mitotic cell division were strongly represented in the set of genes upregulated upon TDRD9 depletion (Figure 5C). RNA binding categories were also enriched when Molecular function-GO enrichments were analyzed (Figure 5D). Pathways such as M phase (Bonferroni adjusted *P* = 0.0005), DNA replication (*P* = 0.0008), metabolism of RNA (*P* = 0.0013) and mitotic cell cycle (*P* = 0.0029) from the Pathway Commons database [27] were also enriched among the upregulated genes. Table 1 summarizes the effect of siTDRD9-1 or siTDRD9-2 on the transcript level of several cell cycle and DNA repair genes. Thus, genes whose expression normally increases at the S or G2/M phases, including those that encode subunits of the condensin and cohesin complexes (*SMC1A, SMC2, SMC3* and *ESCO1*), centrosomal structural components (CEP250, CEP290, and CEP350), cell cycle regulators (*CCND2, CDC27,* and *CDCA2*), and the proliferation marker Ki-67 (*MKI67*), were upregulated upon TDRD9 depletion. Supplementary Figure 8 shows that expression of cyclin D2 gene (*CCND2*) was also increased in other two lung cancer cell lines: H1299 and H441. In summary, transcriptomic analysis together with the cell cycle and proliferation data indicate that a decrease of *TDRD9* expression levels causes a cell cycle arrest in S phase.

**Table 1:** Genes upregulated upon silencing of TDRD9 in H1993 cells

Gene symbol	Gene full name	Fold change	Fold change
		siTDRD9-1	siTDRD9-2
*CEP250*	Centrosomal protein 250kda	4.12	3.75
*MKI67*	Marker of proliferation Ki-67	4.10	3.18
*PRKDC*	Protein kinase, DNA-activated, catalytic polypeptide, DNA-PKcs	4.02	4.13
*ESCO1*	Establishment of sister chromatid cohesion N-acetyltransferase 1	3.23	3.54
*CEP350*	Centrosomal protein 350kda	2.98	2.84
*CDK11A*	Cyclin-dependent kinase 11A	2.91	3.21
*SMC1A*	Structural maintenance of chromosomes 1A	2.89	3.00
*BOD1L1*	Biorientation of chromosomes in cell division 1-like 1	2.79	3.56
*ATRX*	Alpha thalassemia/mental retardation syndrome X-linked	2.76	3.18
*SMC3*	Structural maintenance of chromosomes 3	2.74	2.95
*CDC27*	Cell division cycle 27	2.67	2.76
*SMC2*	Structural maintenance of chromosomes 2	2.54	2.39
*ERCC6L2*	Excision repair cross-complementation group 6-like 2	2.26	2.49
*RIF1*	Replication timing regulatory factor 1	2.15	2.28
*CCND2*	Cyclin D2	2.12	3.16
*PARP1*	Poly (ADP-ribose) polymerase 1	2.11	2.47
*CDCA2*	Cell division cycle associated 2	2.05	2.15
*CEP290*	Centrosomal protein 290kda	2.05	2.15
*MAP4*	Microtubule-associated protein 4	2.03	2.01

### *TDRD9* depletion causes accumulation of DNA damage

**Figure 6:**
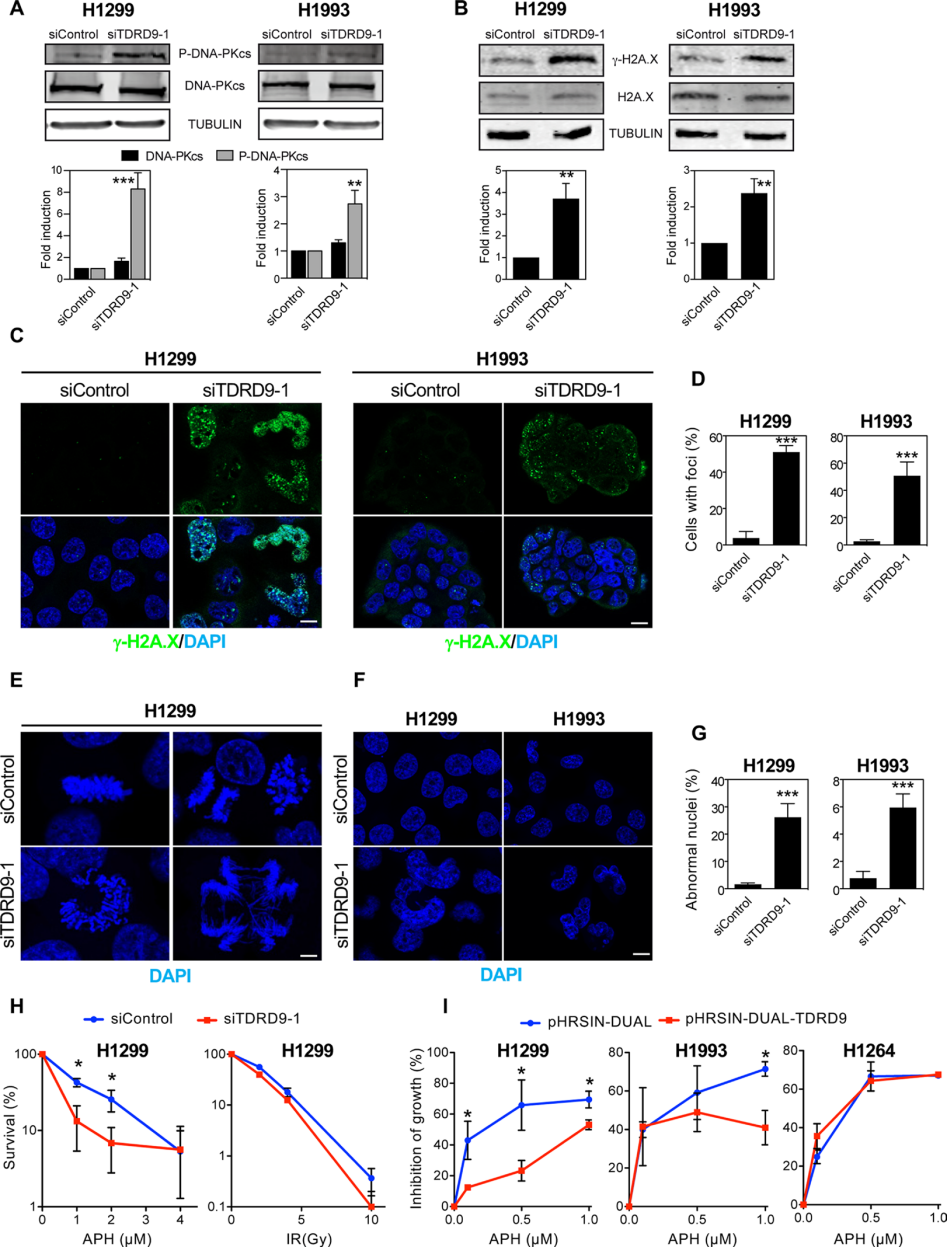
TDRD9 depletion causes DNA damage (**A**, **B**) Effect of *TDRD9* knockdown on the levels of P-DNA-PKcs protein (A) and γ-H2A.X (B) in H1993 and H1299 cell lines. Proteins levels were measured by Western blot using antibodies against the indicated proteins, 72 hours after the transfection of siRNA control (siControl) or siRNA against *TDRD9* (siTDRD9-1). Protein levels were quantified by Odyssey imaging and plotted (lower panels). Intensity values were normalized to the loading control α-tubulin (A) or to the non-modified H2A.X (B). (**C**) Immunofluorescence of γH2A.X, 72 hours after the transfection of siControl or siTDRD9-1 in H1299 and H1993 cell lines. Nuclear DNA was counterstained with DAPI. (**D**) Quantification of γH2A.X-positive cells. Only nuclei with more than two foci were recorded. Data shown are means of three independent experiments. Error bars represent ± SD values. (**E**) Aberrant mitosis are observed in H1299 cells 72 hours after siTDRD9-1 transfection. Nuclei were stained with DAPI (blue). (**F**) Abnormally-shaped nuclei (arrow) observed in H1299 and H1993 cell lines 72 hours after siTDRD9-1 transfection. Nuclei were stained with DAPI (blue). **C**, **E**, **F**. Bars, 10 μm (**G**) Quantification of the percentage of abnormal nuclei observed in H1299 and H1993, 72 hours after siTDRD9-1 transfection as compared to cells transfected with siControl. (**H**) Survival curves (clonogenic assay) in response to increasing doses of aphidicolin (left panel) or ionizing radiation (right panel) in H1299 cells transfected with siControl or siTDRD9. (**I**) Growth inhibition by sublethal concentrations of aphidicolin of H1299, H1993 and H1264 cells transduced with lentiviral particles expressing TDRD9 (pHRSIN-DUAL-TDRD9) or control particles (pHRSIN-DUAL). Student’s *t*-test ^*^P < 0.01; ^***^P < 0.001; ^***^P < 0.0001.

Expression of several genes involved in the DNA damage response was increased upon TDRD9 depletion (Table 1). In particular, the expression of *PRKDC,* which encodes the catalytic subunit of the DNA activated protein kinase (DNA-PKcs), was upregulated in *TDRD9*-silenced cells. DNA-PK is a central regulator of the response to double strand breaks that catalyzes the phosphorylation of histone H2A.X on Ser139 (γH2A.X). γH2A.X helps to recruit repair factors to double strand breaks [28] and coordinates the signal transduction cascades required for efficient repair [29]. Furthermore, DNA damage often blocks the progression of the DNA replication machinery and causes S-phase arrest, a phenotype observed in TDRD9-depleted cells. Therefore, we decided to investigate whether a reduction in the level of TDRD9 causes an increase of double-strand breaks (DSBs), as manifested by an increased level of activated DNA-PKcs (phospho-DNA-PKcs) and γH2A.X signals. As shown in Figure 6, siTDRD9-1-treated H1993 and H1299 cells presented increased levels of phospho-DNA-PKcs, as determined by Western blotting (Figure 6A). Consistently, a strong increase in the γH2A.X signal was also detected by both Western blotting (Figure 6B) and immunofluorescence (Figure 6C and 6D) in both cell lines. We also observed that TDRD9-depleted cells presented frequent aberrant mitosis and DNA bridges during anaphase (Figure 6E), indicative of a defective chromosomal segregation, which can originate from defects in DSB repair [30]. Finally, frequent abnormally-shaped nuclei were also found (Figure 6F and 6G), which is also a hallmark of defective chromosomal segregation [31, 32]. Therefore, we hypothesized that TDRD9 protects cells from DNA damage in TDRD9-positive lung carcinoma cells. Replicative stress is a common source of DNA damage in tumor cells [33]. In order to investigate whether TDRD9 is related to replicative stress, we have determined the sensitivity of TDRD9-depleted cells to the replication stress inducer aphidicolin. Cells treated with siTDRD9.1 were hypersensitive to aphidicolin as compared to control cells (Figure 6H left panel). The non-linearity in the clonogenic survival plot of data from depleted-cells (Figure 6H, red) is indicative of the existence of a mixed population, probably as a consequence of differential silencing of *TDRD9* in the siTDRD9-treated culture. In contrast, TDRD9-depleted cells were not hypersensitive to ionizing radiation (Figure 6H right panel). We next investigated whether an additional increase of the amount of TDRD9 protected cells from replicative stress caused by aphidiolin. Interestingly, overexpression of TDRD9 in the TDRD9-positive H1299 and H1993 cell lines increased resistance to the drug (Figure 6I). However, TDRD9 overexpression did not have any significant effect in the TDRD9-negative cell line H1264 (Figure 6I). Taken together, our data suggest that TDRD9 plays a role in protecting a subset of lung cancer cells from replicative stress.

## DISCUSSION

Here we show that the hypomethylation of the CpG island of *TDRD9* leads to its expression in a subset of LA and NSCLC tumors and cell lines. We found that *TDRD9* expression is associated to poor prognosis for persons with LA. Importantly, knockdown of *TDRD9* in TDRD9-positive cell lines causes a decrease of cell proliferation, cell cycle arrest, increased DNA damage and apoptosis, indicating that TDRD9 could be a potential therapeutic target for TDRD9-positive tumors.

TDRD9 has been previously identified as a putative Cancer/Testis antigen (CTA), in a microarray-based transcriptomic analysis of colorectal tumors [34]. CTAs are a group of tumor-associated antigens that are normally expressed in adult testis and often aberrantly expressed in several types of cancers [13–15]. Therefore, these data suggest that *TDRD9* gene is also highly expressed in a subset of colorectal tumors. As we show here for *TDRD9*, CTA genes expression in tumors correlates with hypomethylation of their promoter-associated CpG islands [35]. Expression of testis-specific genes in tumor cells is often explained as a consequence of the dedifferentiation process that occurs during cell transformation [15]. Therefore, we hypothesize that *TDRD9* hypomethylation and de-repression may be accompanied by de-repression of other CTA genes. However, expression of *TDRD9* is not correlated with that of *MAGEA1*, *MAGEA2*, *MAGEA3*, or *MAGEA4* (Supplementary Figure 9), which are CTA genes often expressed in lung cancer and melanomas [36]. In contrast, expression of *MAGEA1-4* genes correlates positively. These data indicate that hypomethylation and de-repression of *TDRD9* are not a consequence of a general de-differentiation and hypomethylation of all CTA genes. We have also shown that *TDRD9* hypomethylation does not correlate with a widespread hypomethylation of the chromosomal region that contains the *TDRD9* gene. Therefore, we speculate that hypomethylation of the *TDRD9* CpG island is a specific tumor type-dependent event that is favored by the selective advantage conferred by the expression of TDRD9. Interestingly, forced hypomethylation and de-repression of *TDRD9* by 5-aza-2´-deoxycytidine treatment of TDRD9-negative cells, does not promote a stable de-repression of the gene, suggesting that TDRD9 expression does not confer a general selective advantage for all lung carcinoma tumor cells.

Interestingly, SPN-E (the *Drosophila* homologue of TDRD9) and some other factors involved in piRNA metabolism, such as PIWI and AUB, are ectopically expressed in malignant brain tumors in *Drosophila* [37]. Several studies have reported aberrant and ectopic expression of PIWIL proteins in human tumors of different origins [38–41]. However, very little is known about the mechanism by which PIWIL proteins contribute to tumor cells growth. One obvious possibility is that PIWIL proteins control piRNA metabolism and transposon silencing in tumor cells; however, there is no evidence for this statement. Wang et al., have recently reported that ectopic expression of *PIWIL4* in breast cancer does not affect significantly piRNA biosynthesis [41]. The strongest molecular phenotype of *Tdrd9*-/- mutant mice is the massive de-repression and hypomethylation of *Line-1* [2]. In contrast, we have observed no correlation between either *TDRD9* expression or *TDRD9* depletion and the level of *LINE-1* transcription. Taken together, these data suggest that the TDRD9 function in tumor cells is not related to control of *LINE-1* expression. Therefore, how does TDRD9 expression favor cancer cell proliferation? We have found that *TDRD9* silencing in TDRD9-positive cell lines caused a strong increase of γH2A.X, a chromatin marker of DSBs. TDRD9-depleted cells were not hypersensitive to γ-irradiation, suggesting that TDRD9 is not involved in the DSB repair process. However, TDRD9-depleted cells presented problems in S phase progression and exhibited hypersensitivity to the replication stress inducer aphidicolin. In addition, overexpression of TDRD9 protected from aphidicolin treatment in TDRD9-positive cell lines but not in the TDRD9-negative cell line H1264. Aphidicolin is a replication inhibitor that causes the replication folk to stall [42], activates the DNA replication checkpoint and increases DSBs [43]. It is well-established that replication stress is a hallmark of cancer and generates genetic instability [33]. We propose that TDRD9 plays a role in protecting a subset of lung carcinoma tumor cells from replicative stress. The mechanism by which TDRD9 affects replicative stress is unknown. TDRD9 is a putative RNA helicase that contains a RNA binding domain and a TUDOR domain, the latter of which interacts with several RG and RGG motif-containing proteins (our unpublished results). Most RG/RGG motif proteins are involved in RNA biogenesis and processing [44]. In addition, several genes related to RNA metabolism were upregulated upon TDRD9 depletion, including *DDX6, PAPOLA, DHX9, XRN1, SF3B1, FMR1,* and *ILF3* (see Supplementary Table 1). Many RNA metabolism factors affect genome stability [45], making it therefore possible that interactions created by the ectopic expression of TDRD9 in cancer cells contribute to alleviate replication stress.

In conclusion, our data support that TDRD9 is not only a prognosis marker in lung cancer and potentially in other types of cancer (melanoma), but also a possible therapeutic target for TDRD9-positive tumors by inhibiting its RNA helicase enzymatic activity or by blocking its TUDOR domain with specific drugs.

## MATERIALS AND METHODS

### Cells, culture media, and treatments

The NCI-H1299, NCI-H1975, NCI-H1993, NCI-H441, NCI-H23, NCI-H1264, and NCI-H522 cell lines were grown in RPMI medium; the A549 and A427 cell lines were grown in DMEM/F12 medium; and the HeLa cell line was grown in DMEM medium. In all cases, media was supplemented with 10% fetal bovine serum, 100 units/mL penicillin and 100 μg/mL streptomycin. Cells were maintained at 37°C in 5% CO_2_. Experiments were performed in less than 20 passages cells. Cell lines were obtained from the M. Esteller Laboratory (IDIBELL, Barcelona). Cells were periodically (ones per year) checked for *Mycoplasma* contamination and infected stocks were discarded. For *in vivo* demethylation assays cells were treated with 5-aza-2´-deoxycytidine (0.5 µM) for 72 hours.

### DNA methylation analyses

DNA methylation profiles were obtained by bisulphite genomic sequencing of at least eight clones. Genomic DNA was first modified with bisulphite-mediated conversion of unmethylated cytosines to uracil and then purified using the EZ DNA Methylation-Gold Kit (ZYMO Research). The resulting modified DNA was amplified by polymerase chain reaction (PCR) using the primers listed in Supplementary Table 2. The resulting amplified products were gel-purified, subcloned into the pGEM-T Vector Systems (Promega), and sequenced using the T7 and SP6 primers.

### *TDRD9* silencing by siRNAs

For *TDRD9* silencing, the different cell lines were transiently transfected with siRNAs using Oligofectamine (Invitrogen) for 72 hours. The following siRNA sequences were used: siTDRD9-1: 5´-GCAACUUGGUAAACUCAUA-3´; siTDRD9-2: 5´- AGCGCACCAUCCUUCUACUA-3´; and siControl: 5´ -CGUACGCGGAAUACUUCGA-3´.

### Antibodies, western blotting, and immunofluorescence

Western blotting and immunofluorescence were performed as previously described [46]. The antibodies used were: rabbit polyclonal anti-phospho-DNA-PKcs (Ser2056) (4215, Cell Signaling), mouse monoclonal anti-phospho-H2A.X (Ser139) (05-636, Millipore), and mouse monoclonal anti-alpha-Tubulin (T9026, Sigma-Aldrich). As secondary antibodies for Western blotting, goat IRDye 680RD anti-mouse IgG (H+L) (926-68070, Li-COR Bioscience), and IRDye 800CW anti-rabbit IgG (H+L) (926-32211, Li-COR Bioscience) were used. Results were visualized using an Odyssey Infrared Imaging System (Li-COR Bioscience). Alexa Fluor 488 goat anti-mouse IgG (A11001, Life Technologies) was used as a secondary antibody for immunofluorescence.

### Lentivirus production and transduction assays

For lentiviral production, 2 × 10^6^ HEK293T cells were transfected with Fugene 6 (Promega) using 7.5 *μ*g of the transfer vectors pHRSIN-DUAL with 5 and 2.5 *μ*g of the packaging plasmids pCMVDR8.91 and pVSVG, respectively. Lentiviruses were harvested 72 h posttransfection, passed through a 0.45-*μ*m filter and concentrated by ultracentrifugation at 100 000 × *g* for 90 min. Virus particles were resuspended in RPMI medium, snap frozen in liquid nitrogen and stored at −80 °C. Titers of pHRSIN-DUAL lentiviral particles were determined by FACS analysis of GFP-positive H1993 infected cells. For ectopic expression of TDRD9 cells were infected with pHRSIN-DUAL or pHRSIN-DUAL-TDRD9 lentivirus (MOI = 2), respectively. The dual-promoter lentivector pHRSIN-DUAL (also known as pHRSIN-CSGWdINotI_pUb_Em) was kindly provided Mary K Collins (Windeyer Institute, London). For construction of the pHRSIN-DUAL-TDRD9 vector a cDNA encoding human TDRD9 was provided by RZPD, Berlin, Germany (I.M.A.G.E. Consortium (LLNL) cDNA clones) (RZPD clone ID:IMAGp998G0610753Q). BamHI restriction sites were inserted before the ATG of *TDRD9* cDNA sequence using standard PCR techniques. A *Bam*HI-*Not*I fragment containing the full length TDRD9 cDNA was cloned into pHRSIN-DUAL lentiviral vector previously linearized with *Bam*HI and *Not*I.

### Cell cycle and apoptosis analysis using flow cytometer

Cells were harvested, washed with PBS and resuspended in ice-cold PBS. Ethanol (70%) was added dropwise while vortexing at low speed, and cells were then fixed at 4°C for at least 1 hour. Cells were washed with PBS and treated with FACS buffer (250 μg/mL RNase A [Sigma-Aldrich], and 10 μg/mL propidium iodide diluted in PBS). Cells were incubated at 37°C for 30 minutes and then analyzed using a FACSCalibur (BD). Apoptosis was determined by measuring the percentage of cells containing a subG1 DNA content.

### Clonogenic survival and growth inhibition assays

For clonogenic survival assays, siRNA-treated cells were plated in triplicate on 10-cm dishes at clonal density (1000-2000 cells), allowed to adhere for 8 h, and damage treatments administered (aphidicolin or γ-irradiation). After 8–10 days of growth, plates were rinsed, fixed/stained in 20% ethanol/4% crystal violet (w/v), rinsed in distilled water and colonies counted. Results were normalized respect to untreated conditions to adjust for plating efficiency and determine percentage survival.

For growth inhibition assays, 24 h after lentiviral transduction cells were treated with sublethal concentrations of aphidicolin (0.1 µM, 0.5 µM and 1 µM) and allowed to grow for additional 48 h. Then cells were counted and % of growth inhibition respect to untreated cells was calculated.

### RNA extraction and mRNA quantification

Total RNA was isolated by using the RNeasy Kit (Qiagen) following manufacturer’s instructions, including DNase I digestion to avoid potential contaminations of DNA. The cDNA was generated from 100 ng of total RNA by using SuperScript First Strand Synthesis System (Invitrogen). cDNA (2 µl) solution was used as a template for conventional or real-time quantitative PCR (RT-PCR or RT-qPCR, respectively). Gene products were quantified by qPCR with the Applied Biosystems 7500 FAST Real-Time PCR System, using Applied Biosystems Power SYBR Green Master Mix. Values were normalized to the expression of the human *GAPDH* housekeeping gene. Each experiment was performed at least in triplicate. Sequences of all oligonucleotides used are listed in Supplementary Table 2.

### Microarray expression analysis

Three independent experiments of transfection of H1993 cells with siControl, siTDRD9-1 or siTDRD9-2 siRNAs were performed. After 72 hours, total RNA was isolated from cells using RNeasy Mini Kit (Qiagen). Purity and quality of isolated RNA were assessed by RNA 6000 Nano assay on a 2100 Bioanalyzer (Agilent Technologies, Santa 6 Clara, CA). RNA (100 ng) was used to produce end-labelled biotinylated ssDNA. Labeled ssDNA was hybridized to the GeneChip^®^ PrimeView Gene Expression Array oligonucleotide microarray (Affymetrix, Santa Clara, CA) according to manufacturer’s recommendations. Arrays were scanned using the GeneChip Scanner 3000 7G (Affymetrix), and raw data were extracted from the scanned images and analyzed with the Affymetrix GeneChip Command Console Software (Affymetrix). The raw array data were normalized using the Robust Multichip Average (RMA) method [47]. Fold-change and statistic parameters of siControl versus siTDRD9-1 or siTDRD9-2 comparisons were performed using the LIMMA package [48] through oneChannelGUI [49]. Genes differentially expressed more than 2-fold (lineal change) and with FDR < 0.01 were selected. Genes that were differentially expressed with both siRNAs that target *TDRD9* transcript were then selected for further analysis. Gene ontology functional categories were analyzed using DAVID [50] or WebGestalt [51]. The significance of the enrichment was computed using the hypergeometric test. Pathways enrichment of the Pathway Commons database was screened using the WebGestalt software packages. Bonferroni-adjusted *P* values of the hypergeometric test were used to determine enrichment significance. Microarray data are available from the GEO database (accession number GSE104151).

### Analysis of tumor datasets and statistics

Microarray gene expression data were obtained from ONCOMINE [16]. *P* values were calculated using a two-tailed Student´s *t*-test with a confidence interval of 95% by using GraphPad *Prism* version 5.0. Shapiro-Wilk tests for normality were performed in http://scistatcalc.blogspot.com.es/2013/10/shapiro-wilk-test-calculator.html. RNA-seq expression data were obtained from The Cancer Genome Atlas (TCGA) (http://cancergenome.nih.gov/). Clinical data of the cohorts for lung adenocarcinoma (TCGA-LUAD) [21] and skin melanoma (TCGA-SKCM) [22], and DNA methylation data, were obtained through cBioPortal (http://www.cbioportal.org/) [23]. Regression plots, and determination of Pearson coefficients were performed using Excel version 15.24. Survival plots and the log-rank test were performed using GraphPad *Prism* version 5.0. NIH Roadmap Epigenomics Mapping Consortium CpG methylation data were visualized by using the UCSC Genome Browser (http://genome.ucsc.edu/).

## SUPPLEMENTARY MATERIALS FIGURES AND TABLES




