# Supplementary material for: Expression of TDRD9 in a subset of lung carcinomas by CpG island hypomethylation protects from DNA damage
Source: Oncotarget. 2017 Nov 27;9(11):9618–31. doi: 10.18632/oncotarget.22709 (PMC5839389; doi:10.18632/oncotarget.22709)
Supplement: Supplementary file 1 [file oncotarget-09-9618-s001.pdf]

Expression of TDRD9 in a subset of lung carcinomas by CpG island hypomethylation protects from DNA damage

SUPPLEMENTARY MATERIALS

Supplementary Table 1: Genes differentially regulated after knockdown of TDRD9 with siTDRD9-1 or siTDRD9-2 in H1993 cells. Only genes misregulated with both siRNAs are shown. See Supplementary_Table_1.

Supplementary Table 2: Oligonucleotides list

Loci	Forward 5´-3´	Reverse 5´-3´	Used for
TDRD9	TCAAGAGAATTAGAGAGG	GGAATGTGTTTCAACACG	RT-PCR
PIWIL1	TGGTTATATTCAGCCTAGGC	CTTCTGGCTTCCATCAGTGG	RT-PCR
PIWIL2	CACTCCTGGAACTGTGGTAC	CTCACCAGCCCATCTCCAAG	RT-PCR
PIWIL3	GTTCAGAGGGTACAGTGGTAC	CGGTATAACTGAATGGCCTTC	RT-PCR
PIWIL4	CTTGGGAACAAGCAGGATCTC	GGATGGCACCGTCGAATGC	RT-PCR
UTR-LINE1	GAATGATTTTGACGAGCTGAGAGAA	GTCCTCCCGTAGCTCAGAGTAATT	RT-qPCR
TDRD9	TGTCTTGGGCCAGAGAGAGT	TAGGGCTTTTCATGCCACTT	RT-qPCR
GAPDH	GAGTCAACGGATTTGGTCGT	AATGAAGGGGTCATTGATGG	RT-PCR and RT-qPCR
CCND2	GCCACCGACTTTAAGTTTGC	GCTCACTTCCTCATCCTGCT	RT-qPCR
AXL	CCAGCACCTGTGGTCATCT	CATCTGAGTGGGCAGGTACA	RT-qPCR
CTGF	GCAGGCTAGAGAAGCAGAGC	TGGAGATTTTGGGAGTACGG	RT-qPCR
CTSV	GGGAAACATGGCTTCACAAT	ACAGAGGCTCACGGAACACT	RT-qPCR
QKI	AACTTCTGCGGGATCTTCAA	ATAGGTCCCACAGCATCAGG	RT-qPCR
PBX2	AAGACATCGGGGACATTCTG	AAGAGAGCAGGCTTCATTCG	RT-qPCR
HOXB8	GGAGCTGGAGAAACAGAAGC	CGGAGATGGAGCAAGTCTTC	RT-qPCR
RASSF8	GGTCAAAGGGGAGATTGACA	GCCGCAACTCCTTAGTCAAC	RT-qPCR
PRKDC	CCGGACGGACCTACTACGACT	AGAACGACCTGGGCATCCT	RT-qPCR
TDRD9	TGAGGTTTTAGAAAGGTTTATTTTG	CCAACAACTCCACATTAATCAC	CpG island amplification
28S	CTGGAGAGGCCTCGGGATCC	TACCCACCCGACCCGTCTTG	RT-qPCR

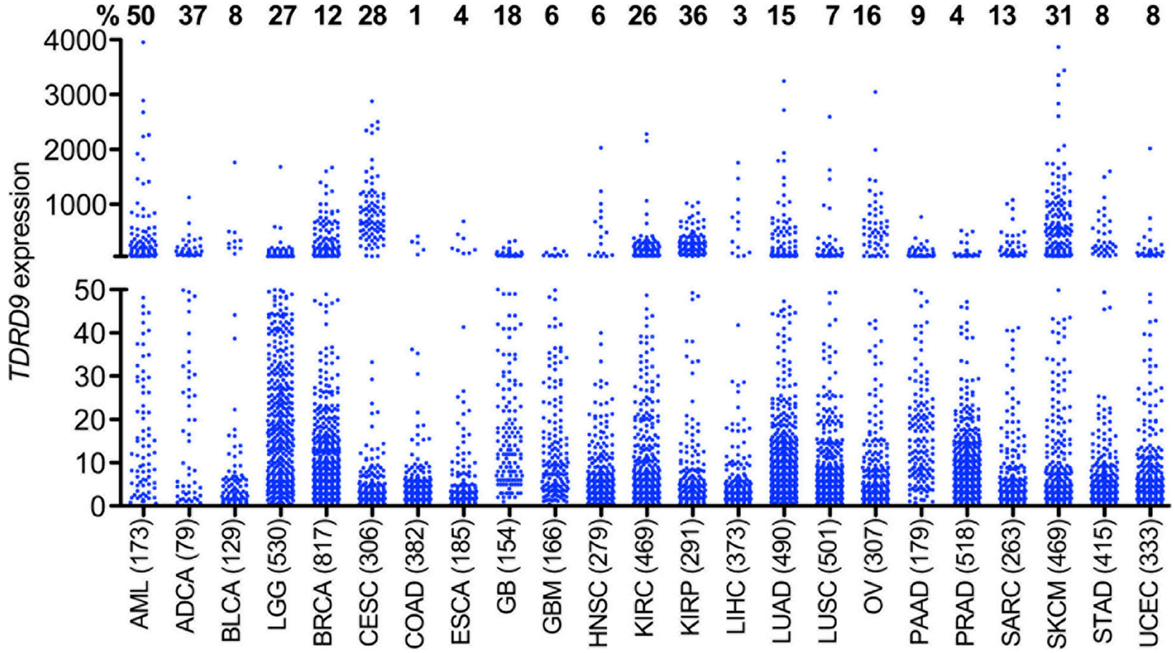

Supplementary Figure 1: Levels of *TDRD9* transcript in tumors of different types from TCGA. RNA-seq expression data were obtained from TCGA. Upper numbers are the percentage of tumors with an expression level > 50 (RSEM normalized) for each type of tumor. Abbreviations of the tumors are: AML, acute myeloid leukemia; ADCA, adrenocortical carcinoma; BLCA, bladder urothelial carcinoma; BRCA, breast invasive carcinoma; CESC, cervical squamous cell carcinoma and endocervical adenocarcinoma; COAD, colon adenocarcinoma; ESCA, esophageal carcinoma; GB, glioblastoma; GBM, glioblastoma multiforme; HNSC, head and neck squamouse cell carcinoma; KIRC, kidney renal clear cell carcinoma; KIRP, kidney renal papillary cell carcinoma; LGG, brain lower grade glioma; LIHC, liver hepatocellular carcinoma; LUAD, lung adenocarcinoma; LUSC, lung squamous cell carcinoma; OV, ovarian serous cystadenocarcinoma; PAAD, pancreatic adenocarcinoma; PRAD, prostate adenocarcinoma; SARC, sarcoma; SKCM, skin cutaneous melanoma; STAD, stomach adenocarcinoma; UCEC, uterine corpus endometrial carcinoma. Number of tumors in each cohort is shown between braquets.

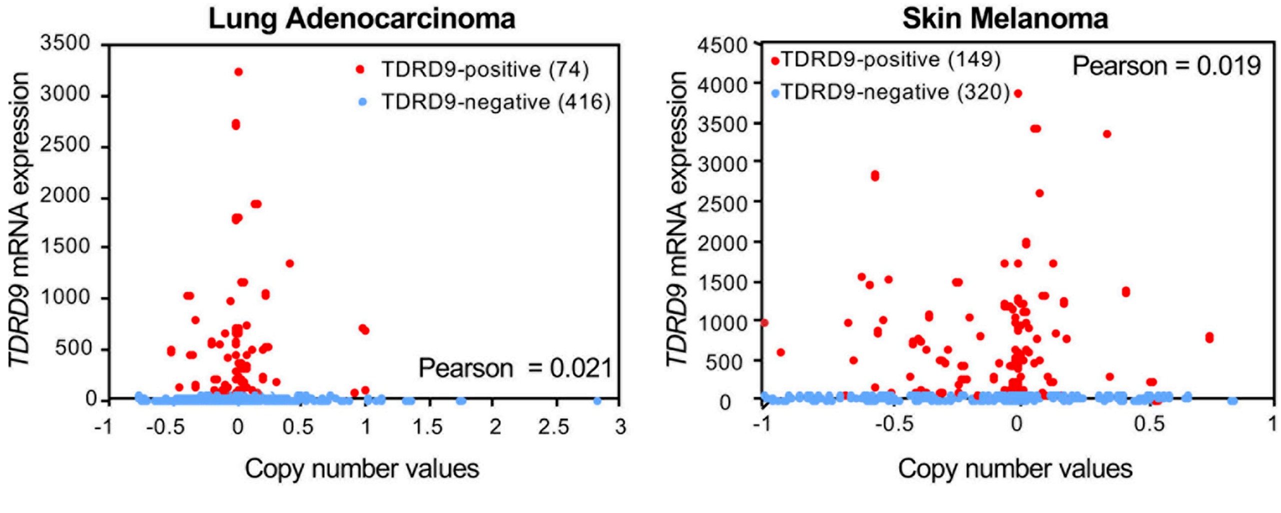

Supplementary Figure 2: Scatter diagram of copy number versus *TDRD9* gene expression values. The horizontal axis represents *TDRD9* copy number, and the vertical axis expression of TDRD9 mRNA (RNA-seq data, RSEM normalized), in TCGA lung adenocarcinomas (TCGA-LUAD) and in TCGA skin melanomas (TCGA-SKCM). Copy number values are TCGA level 3 normalized data, with value 0 representing the diploid state of the region.

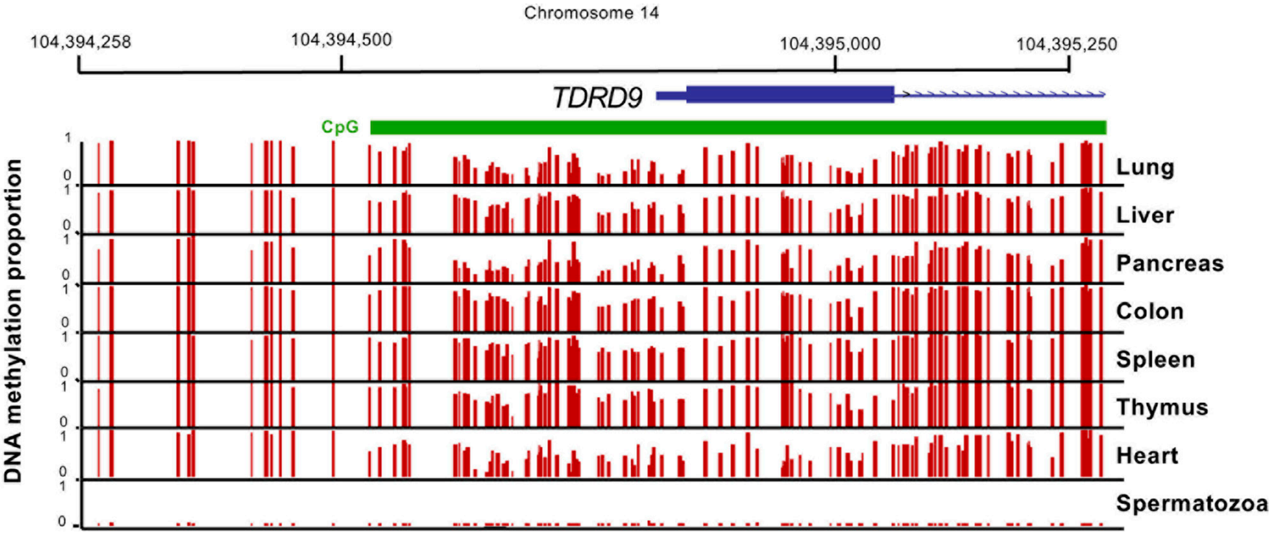

Supplementary Figure 3: DNA methylation levels of the CpG island in the *TDRD9* promoter from different organs. Screenshot from UCSC Genome Browser of the *TDRD9* CpG island region showing NIH Roadmap Epigenomics Mapping Consortium CpG methylation data. Fraction of DNA methylation of each CpG pair between 0 (unmethylated) and 1 (fully methylated) is provided.

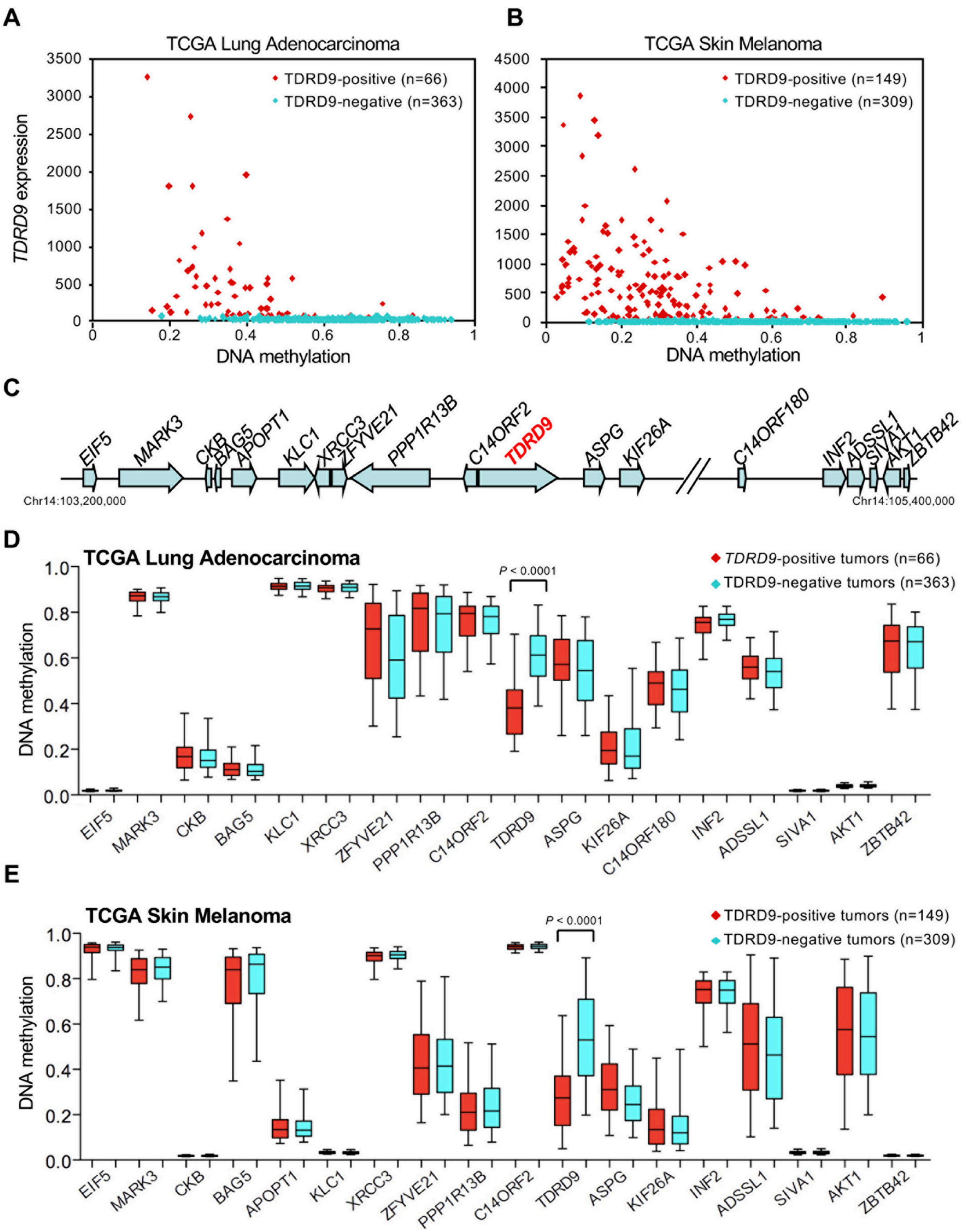

Supplementary Figure 4: Correlation between expression and methylation levels of the *TDRD9* gene. *TDRD9* gene expression (RNA-seq data) was associated to hypomethylation of the *TDRD9* CpG island in the TCGA lung adenocarcinoma (**A**) and the TCGA skin melanoma cohorts (**B**). Spearman correlation test, $r = -0.44$, $P < 0.00001$ for lung adenocarcinoma and $r = -0.54$, $P < 0.00001$ for skin melanoma. TDRD9-positive tumors are presented in red, and TDRD9-negative tumors are shown in blue. (**C**). Scheme showing gene distribution of the *TDRD9* chromosomal region. (**D**, **E**). Boxplots of the methylation levels in 17 genes surrounding *TDRD9* in TDRD9-postive (red) and TDRD9-negative (blue) TCGA lung adenocarcinomas (D) or TCGA skin melanomas (E). Student's *t*-test *P* value is provided when significant. For A, B, D and E, DNA methylation data are provided as the fraction of methylation, which ranges between 0 (unmethylated) and 1 (fully methylated). Significant differences were only observed for the *TDRD9* gene.

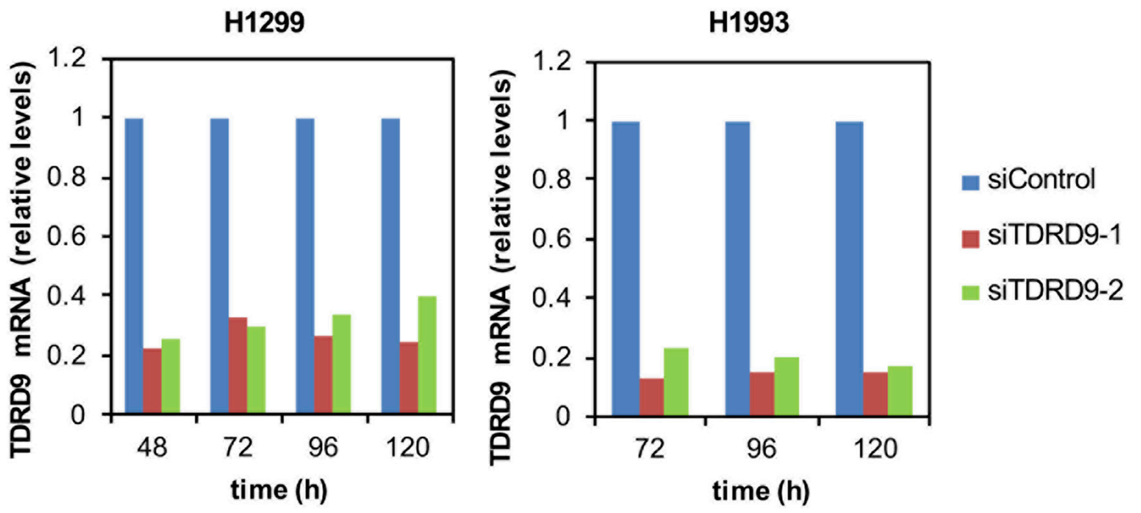

Supplementary Figure 5: Expression levels of *TDRD9* in H1299 and H1993 cell lines after the transfection of the indicated siRNAs at the indicated time points of the growth curves shown in figure 4B. *TDRD9* mRNA levels were determined by RT-qPCR. Data are the mean of $n = 6$ qPCR reactions from two independent experiments.

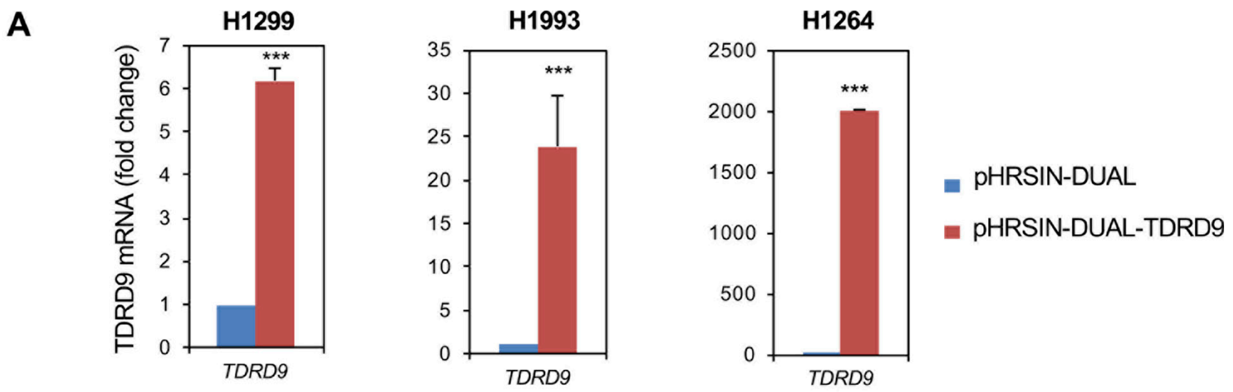

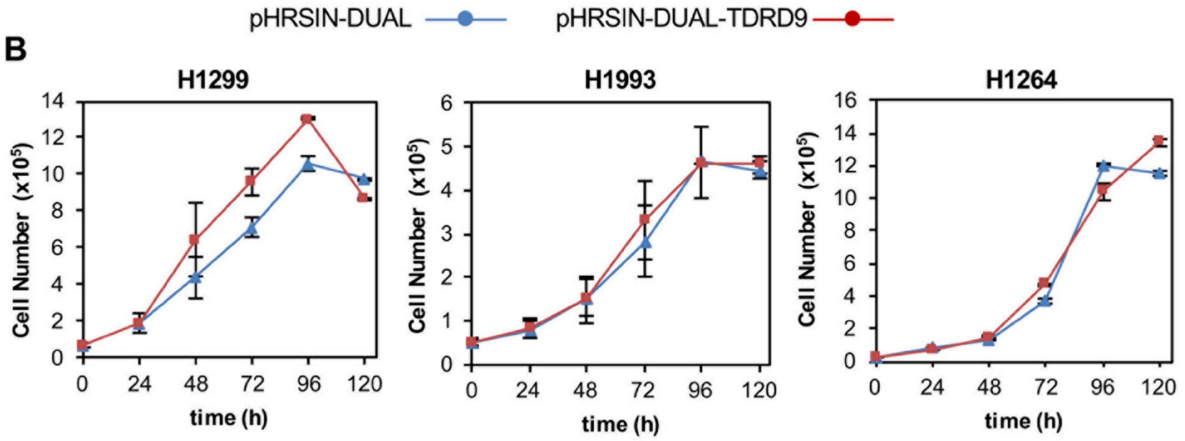

Supplementary Figure 6: Effect of overexpression of *TDRD9* in cell proliferation. (**A**). Levels of *TDRD9* transcript in H1299, H1993 and H1264 cells transduced with TDRD9-expressing lentiviral particles (pHRSIN-DUAL-TDRD9). Empty vector (pHRSIN-DUAL) particles were also transduced as control. mRNA levels were determined by RT-qPCR 72 h after transduction. Data are the mean ± standard error, of at least $n = 6$ qPCR reactions from three independent experiments. Significance respect to the control was tested by using Student's *t*-test. $^{***}P < 0.0001$. (**B**). Growth curve of H1299, H1993 and H1264 cells transduced with control (pHRSIN-DUAL) or TDRD9-expressing (pHRSIN-DUAL-TDRD9) lentiviral particles. Data are the average of three independent experiments. Error bars represent standard error. None of the compared pair of values were significantly different using Student's *t*-test.

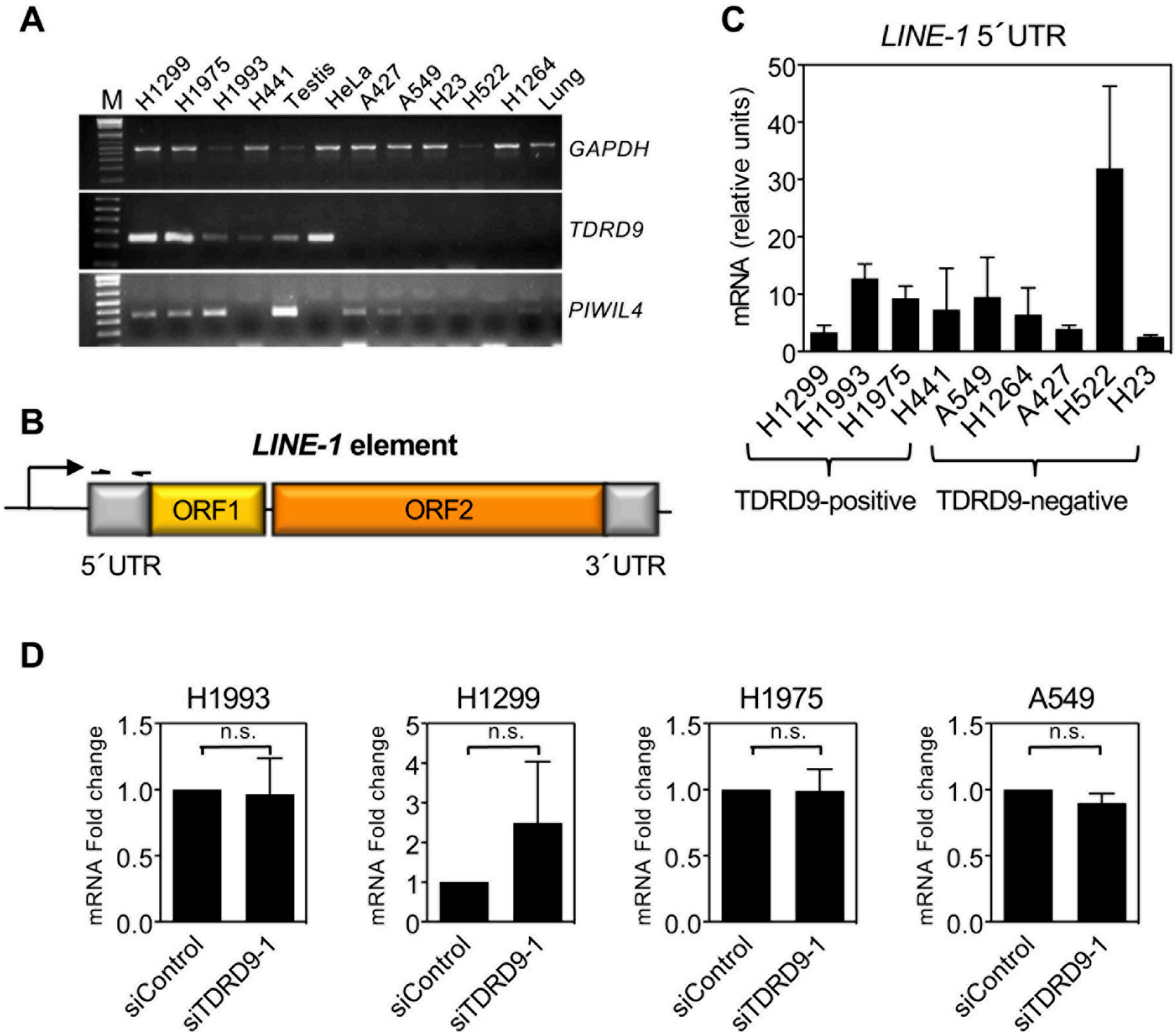

Supplementary Figure 7: TDRD9 does not control *LINE-1* transcript expression in lung cancer cell lines. (**A**) *PIWIL4* expression levels were analyzed by RT-PCR using specific primers. *GAPDH* was used as a loading control and RNA control. The panels corresponding to *GAPDH* and *TDRD9* are the same than in Figure 2A and are included here for comparison. M: 100 bp marker. (**B**). Scheme of *LINE-1* element showing the primers designed for the RT-qPCR, localized in the 5' UTR region. (**C**). Expression level of *LINE-1* element in different TDRD9-postive and TDRD9-negative lung cancer cell lines by RT-qPCR. Data are the mean of at least $n = 6$ qPCR reactions from three independent experiments. Error bars represent ± SD values. (**D**) Effect of the downregulation of TDRD9 expression in the level of *LINE-1* transcript in TDRD9-positive lung cancer cell lines. TDRD9-positive lung cancer cell lines (H1993, H1299, H1975) as well as a TDRD9-negative cell line (A549) were transfected with siControl or siTDRD9-1. The levels of *LINE-1* transcript were determined by RT-qPCR 72 hours after transfection. Data are the mean of at least $n = 6$ qPCR reactions from three independent experiments. Error bars represent ± SD values. None of the compared pair of values were significantly different (n.s.) using Student's *t*-test.

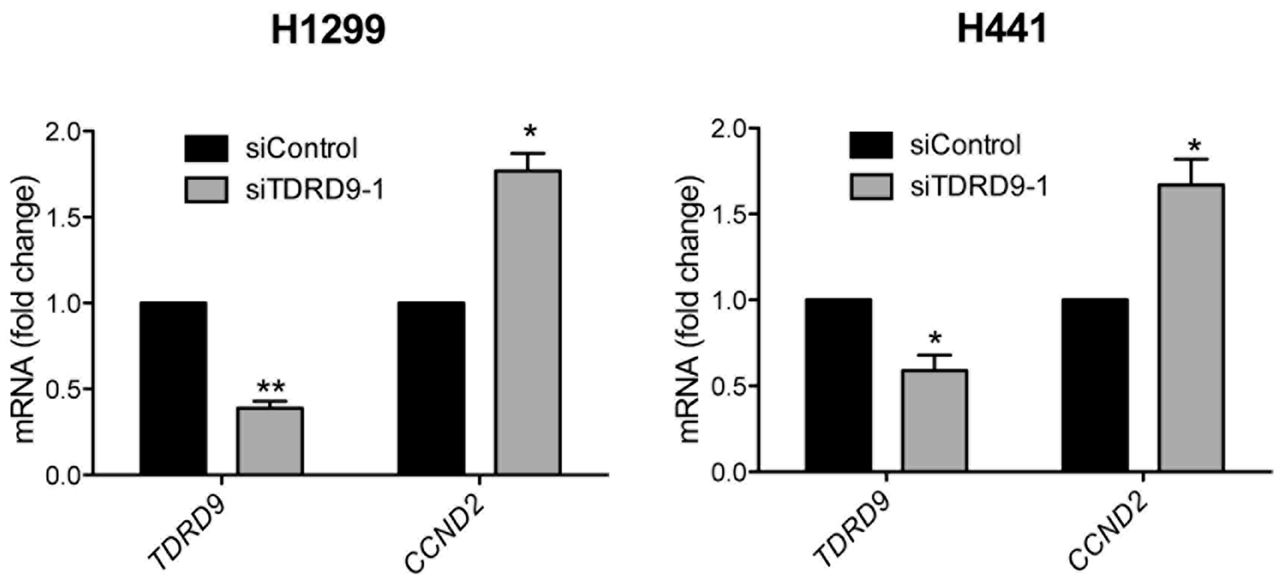

Supplementary Figure 8: Effect of *TDRD9* knockdown on the expression of *CCND2* gene in H1299 and H441 cell lines. Expression levels of *TDRD9* were determined as a control. mRNA levels were determined by RT-qPCR. Data are the mean of at least $n = 6$ qPCR reactions from three independent experiments. Error bars represent standard error. Significance respect to the control was tested by using Student's *t*-test. $^{*}P < 0.01$.

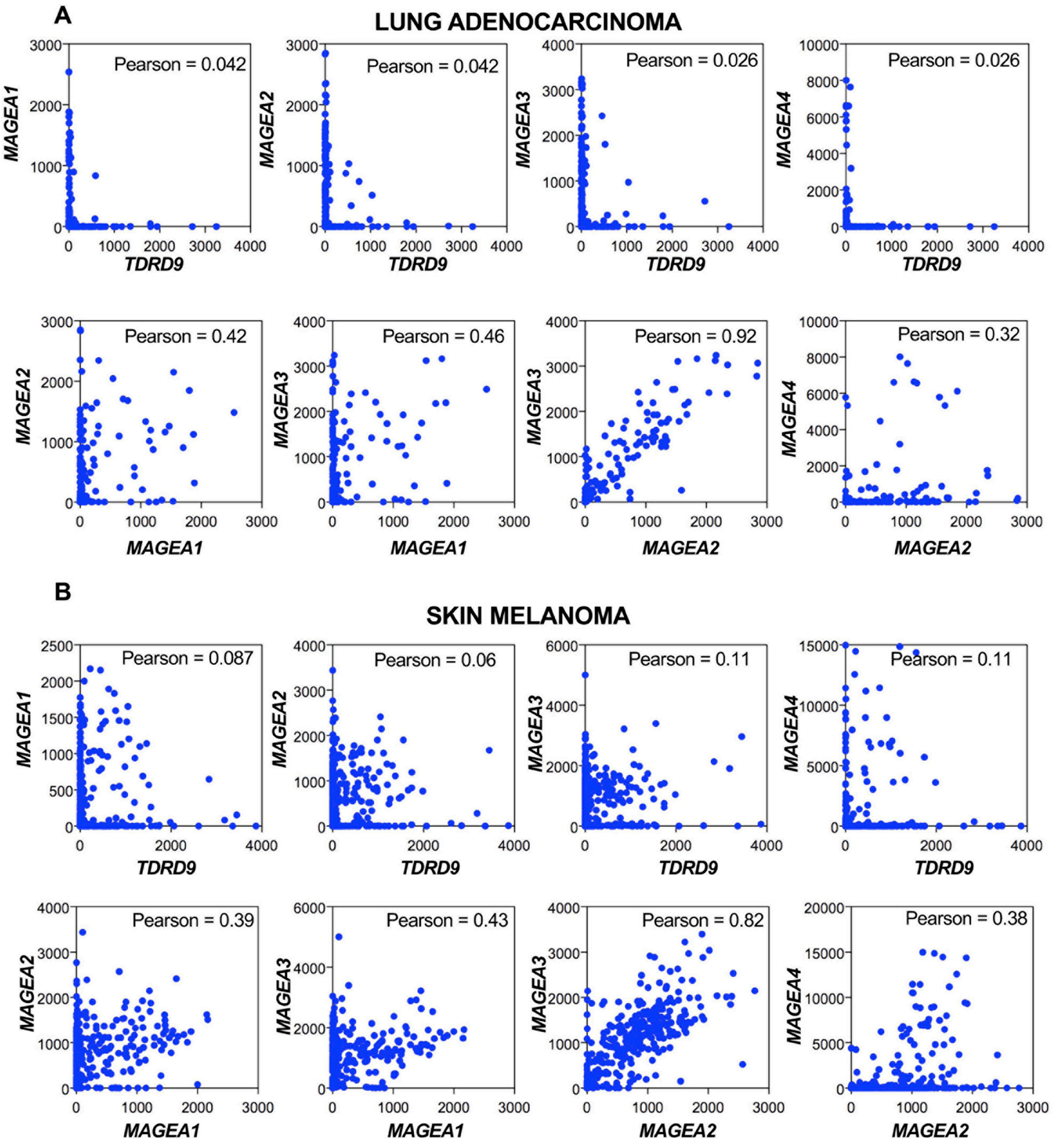

Supplementary Figure 9: Correlation between levels of expression of *TDRD9* and MAGEA genes. Scatter plots showing correlation between levels of expression (RNA-seq data, RSEM normalization) of the indicated genes in the TCGA lung adenocarcinoma (**A**) and the TCGA skin melanoma (**B**) cohorts. Pearson correlation coefficient is shown.
